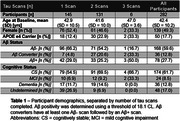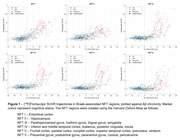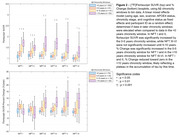# Regional tau burden emerges following beta‐amyloid chronicity timeline in the Down syndrome population

**DOI:** 10.1002/alz70856_106826

**Published:** 2026-01-08

**Authors:** Max McLachlan, Brecca Bettcher, Andrew K McVea, Matthew D Zammit, Lisette LeMerise, Jeremy P. Rouanet, Julie C Price, Dana L Tudorascu, Charles M Laymon, David B. Keator, Patrick J. Lao, Adam Brickman, Tim D Fryer, Sigan L Hartley, Beau Ances, H. Diana Rosas, Sterling C. Johnson, Tobey J. Betthauser, Charles K Stone, Shahid Zaman, Benjamin L Handen, Elizabeth Head, Mark Mapstone, Bradley T Christian

**Affiliations:** ^1^ Waisman Center, University of Wisconsin‐Madison, Madison, WI, USA; ^2^ University of California, Irvine, Irvine, CA, USA; ^3^ Massachusetts General Hospital, Harvard Medical School, Boston, MA, USA; ^4^ University of Pittsburgh, Pittsburgh, PA, USA; ^5^ Department of Neurology, Vagelos College of Physicians and Surgeons, Columbia University, New York, NY, USA; ^6^ Department of Neurology, Vagelos College of Physicians and Surgeons, Columbia University, and the New York Presbyterian Hospital, New York, NY, USA; ^7^ University of Cambridge, Cambridge, United Kingdom; ^8^ Washington University in St. Louis, St. Louis, MO, USA; ^9^ Wisconsin Alzheimer's Disease Research Center, University of Wisconsin School of Medicine and Public Health, Madison, WI, USA; ^10^ Wisconsin Alzheimer's Disease Research Center, University of Wisconsin‐Madison School of Medicine and Public Health, Madison, WI, USA; ^11^ University of Wisconsin‐Madison School of Medicine and Public Health, Madison, WI, USA

## Abstract

**Background:**

Previous work in the Down syndrome (DS) population has revealed early and accelerated accumulation of Alzheimer's disease (AD) pathology when compared with neurotypical adults. Temporal models of [^11^C]PiB PET beta‐amyloid (Aβ) trajectories aligned with the progression of [^18^F]flortaucipir neurofibrillary tau (NFT) burden through Braak‐associated regions (Zammit 2023). These analyses were extended to a larger DS cohort that includes individuals who underwent Aβ imaging with [^18^F]florbetapir. This work investigated the temporal relationship between regional NFT burden and a standardized model of Aβ onset in the DS population.

**Method:**

282 participants with DS underwent longitudinal NFT and Aβ PET imaging (Table 1). PiB and florbetapir scans underwent Centiloid (CL) processing using a previously calibrated pipeline. Flortaucipir scans were realigned, summed 80‐100 min, and warped into standard space. SUVR was calculated (inferior cerebellar grey reference) for Braak‐associated NFT regions (described in Figure 1). Using the sampled iterative local approximation (SILA) method, the average rate of change was discretely sampled across the CL range, yielding a generalized model for amyloid progression. The estimated time‐to‐Aβ onset (or Aβ chronicity) was calculated for each participant by aligning their trajectories to the model. Flortaucipir SUVR and % Change were binned within discrete Aβ chronicity stages and fit to a linear mixed effects model for each region, using:

SUVR or % Change ∼ Age+Sex+Scanner+APOE4+Chronicity Stage+Cognitive Status+(1|Participant)

The model was used to estimate the influence of biological parameters and the relative time of significant NFT onset.

**Result:**

Across all NFT regions, participants with dementia had the highest flortaucipir SUVR and latest chronicity, followed by MCI (Figure 1). SUVR was significantly increased by the 0‐5 years chronicity window in NFT I and II, while NFT III‐VI were not significantly increased until 5‐10 years (Figure 2). % Change was significantly increased in the 0‐5 years chronicity window for NFT I and in the >10 years window for NFT III‐VI. Scanner, cognitive status, and age effects were significant in each region.

**Conclusion:**

This work supports previous findings that NFT regions I‐II demonstrate earlier increases to flortaucipir SUVR than other NFT regions when using a standardized temporal model for Aβ onset.